# Successful implementation of a combined learning collaborative and mentoring intervention to improve neonatal quality of care in rural Rwanda

**DOI:** 10.1186/s12913-018-3752-z

**Published:** 2018-12-04

**Authors:** Jennifer Werdenberg, Francois Biziyaremye, Merab Nyishime, Evrard Nahimana, Christine Mutaganzwa, David Tugizimana, Anatole Manzi, Shalini Navale, Lisa R. Hirschhorn, Hema Magge

**Affiliations:** 1 Partners In Health/Inshuti Mu Buzima, Rwinkwavu, Rwanda; 2 Boston Children’s Hospital, 300 Longwood Avenue, Boston, MA 02115 USA; 3 Harvard TH Chan School of Public Health, 677 Huntington Avenue, Boston, MA 02115 USA; 4 University of Global Health Equity, 800 Boylston St. Suite 300, Boston, MA 02199 USA; 5 University of Rwanda School of Medicine and Health Sciences, PO box 3286, Kigali, Rwanda; 6 Widener University Center for Human and Sexuality Studies, One University Place, Chester, PA 19013 USA; 7 Northwestern University Feinberg School of Medicine, 420 E Superior St, Chicago, IL 60611 USA; 8 Brigham and Women’s Hospital, 75 Francis Street, Boston, MA 02115 USA; 9 Institute for Healthcare Improvement, 20 University Rd, Cambridge, MA 02138 USA

**Keywords:** Quality improvement, Health care system, Children, Quality culture, Developing countries

## Abstract

**Background:**

Globally, neonatal mortality remains high despite interventions known to reduce neonatal deaths. The All Babies Count (ABC) initiative was a comprehensive health systems strengthening intervention designed by Partners In Health in collaboration with the Rwanda Ministry of Health to improve neonatal care in rural public facilities. ABC included provision of training, essential equipment, and a quality improvement (QI) initiative which combined clinical and QI mentorship within a learning collaborative. We describe ABC implementation outcomes, including development of a QI change package.

**Methods:**

ABC was implemented over 18 months from 2013 to 2015 in two Rwandan districts of Kirehe and Southern Kayonza, serving approximately 500,000 people with 24 nurse-led health centers and 2 district hospitals. A process evaluation of ABC implementation and its impact on healthcare worker (HCW) attitudes and QI practice was done using program documents, standardized surveys and focus groups with facility QI team members attending ABC Learning Sessions. The Change Package was developed using mixed methods to identify projects with significant change according to quantitative indicators and qualitative feedback obtained during focus group discussions. Outcome measures included ABC implementation process measures, HCW-reported impact on attitudes and practice of QI, and resulting change package developed for antenatal care, delivery management and postnatal care.

**Results:**

ABC was implemented across all 26 facilities with an average of 0.76 mentorship visits/facility/month and 118 tested QI change ideas. HCWs reported a reduction in barriers to quality care delivery related to training (*p* = 0.018); increased QI capacity (knowledge 37 to 89%, *p* <  0.001); confidence (47 to 89%, p <  0.001), QI leadership (59 to 91%, p <  0.001); and peer-to-peer learning (37 to 66%, *p* = 0.024). The final change package included 46 change ideas. Themes associated with higher impact changes included provision of mentorship and facility readiness support through equipment provision.

**Conclusions:**

ABC provides a feasible model of an integrated approach to QI in rural Rwanda. This model resulted in increases in HCW and facility capacity to design and implement effective QI projects and facilitated peer-to-peer learning. ABC and the change package are being scaled to accelerate improvement in neonatal outcomes.

## Background

Despite declines in under-five mortality in Rwanda and globally, neonatal mortality remains high [1]. Contributors to neonatal mortality include poor coverage and quality delivery of evidence-based interventions known to improve maternal and newborn outcomes [2]. To date, approaches to improve delivery of these interventions have included improving individual provider skills through mentoring and skills-training; health systems strengthening to ensure availability of essential equipment; and quality improvement (QI) interventions, such as learning collaboratives, to drive system change [3, 4].

A learning collaborative brings together QI teams from healthcare facilities to seek improvement in a focused topic area through QI methods and peer learning [5]. Collaboratives often create change packages, collections of high impact change ideas tested during a collaborative, which are used to accelerate quality improvement by spreading potential solutions to common challenges [6–11]. The change package includes change concepts, grouping of similar change ideas into a broader conceptual categories [12].

Building on previous success of mentoring and learning collaboratives [11, 13], the Rwanda Ministry of Health (MOH) in partnership with Partners In Health (PIH), a US-based non-governmental organization, designed and implemented the All Babies Count Initiative (ABC) in 2013. ABC was an 18-month district-wide QI learning collaborative and mentorship effort designed to reduce neonatal mortality by improving health system performance and individual provider behavior to prevent newborn death.

ABC was implemented in two rural Rwandan districts which have been supported by the MOH-PIH partnership since 2005, with additional work starting in 2009 through a health systems strengthening project funded through the Doris Duke Charitable Foundation Africa Health Initiative [14]. The two districts serve a catchment population of approximately half a million and included 24 nurse-led health centers and two district hospitals at the start of ABC [15].

This paper reviews the implementation process and implementation outcomes of the ABC initiative including feasibility and fidelity, acceptability, self-reported changes in health care worker (HCW) attitudes and practice of QI, QI project implementation and the resulting change package.

## Methods

### The ABC intervention

ABC had three primary components. First, based on routine facility data on infrastructure and supplies, baseline gaps in essential neonatal care medical equipment were identified, and essential equipment was provided to close critical needs [16]. Second, utilizing PIH and MOH staff, ABC provided targeted clinical skills training on national neonatal care protocols which included essential newborn care, neonatal resuscitation (using Helping Babies Breathe); and advanced neonatal care for hospital staff. Third, these components were supported by a district-wide 18-month Learning Collaborative organized by ABC mentors with integrated onsite clinical and QI mentorship to improve HCW skills and support system-level QI projects in neonatal and maternal care. Additional program description details can be found elsewhere [17].

The learning collaboratives included four learning sessions during the active collaborative and a final “Harvest Session”. Between the learning sessions, facilities were visited monthly by an ABC mentor who provided individual clinical and team QI mentorship to support improvement in clinical care delivery and in QI project implementation to address identified gaps in the core processes of maternal/newborn health [antenatal care (ANC), delivery management, and postnatal care (PNC)]. ABC mentors were experienced nurses in neonatal care delivery with additional training *PIH Pediatric Program Director* in QI methods, learning collaborative facilitation, data collection and analysis and coaching. To facilitate sustainability, mentoring visits were conducted jointly with existing, district-based MOH mentors whenever possible.

QI teams were formed at each facility, composed of 3–4 strategic team members, usually including a maternity, antenatal, or neonatal charge nurse, the community health officer, and the facility data manager. QI teams were supported to review their performance in eight core indicators and develop QI projects based on the results. QI projects were designed to test specific interventions (change ideas), using standard “Plan, Do, Study, Act” (PDSA) cycles based on the Model for Improvement [18].

Core learning collaborative indicators were chosen through a combination of literature review, expert consultation and review of data available in the Rwandan National Health Management Information System (HMIS) (Table 1).

**Table 1:** ABC Learning Collaborative Core Indicators

Care Domain	ABC Learning Collaborative Indicators	Data Source
Antenatal care	Percent of women completing four standard antenatal care visits (ANC)	HMIS^a^
Delivery	Number of babies with birth asphyxia	HMIS
	Percent of preterm births in which women received antenatal steroids	non-HMIS
	Percent of prolonged rupture of membranes in which women received antibiotics	non-HMIS
	Percent of births at which a skilled birth attendant was available	HMIS
	Time to cesarean section (hospital only)	non-HMIS
Postnatal care	Percent of infants receiving immediate skin-to-skin	HMIS
	Percent of infants checked for danger signs within 24 h of delivery	HMIS

^a^ *HMIS* Health Management Information System

Core indicators included those available from routinely reported data (HMIS) and ones which required review of facility registers (non-HMIS). Non-HMIS indicators were extracted from registers by the mentors and facility QI teams. HMIS indicators were extracted from existing reports with data validation done with facility QI teams by comparing HMIS reports with facility registers. Indicators were collected retrospectively at baseline (minimum 3 months prior to QI project start for non-HMIS indicators and 6 months for HMIS indicators) and monthly during the 18-month implementation. Data were entered into excel databases for review with facility teams, programmatic monitoring and QI activity tracking. A change package for the three care domains of ABC was created at the end of the collaborative based on quantitative and qualitative data collected from the QI projects and participants.

### Data sources

#### Collaborative implementation

Implementation data were collected using routine program monitoring tools. These data included: learning session attendance, duration, and frequency; mentor visit content and frequency; and QI team characteristics.

#### Healthcare worker attitudes and practice of quality improvement

Participant surveys were completed before the first learning session and after the Harvest Session with shorter surveys before other learning sessions. The surveys were adapted from previously used surveys in other quality improvement programs. They included answers on a 5 item Likert scale and open-ended questions used for immediate programmatic management. The scaled questions were included in this analysis. Questions included: confidence in QI methods, leadership, teamwork, data use, peer-to-peer learning, motivation, and work environment using a Likert scale as well as presence of barriers to care such as knowledge, time, and resources.

#### Change package

Change ideas generated and tested by facility teams were captured by mentors using a QI tracking tool. Quantitative pre-post mean analysis of change in the targeted measure was used to identify successful change ideas for inclusion in the change package. During the Harvest Session, each team was asked to rank all tested change ideas using a priority matrix which included: potential impact on health outcomes; volume of patients affected; time to impact; feasibility; and level of other support required. Results of the quantitative analysis and qualitative ranking were used to generate district-wide lists of higher and lower ranked change ideas in each care domain to guide focus group discussions.

During the Harvest Session, one focus group discussion was conducted on each care domain - ANC, PNC and delivery care - with ABC participants in each district for a total of 6 focus group discussions. Discussions were used to capture feedback on the prioritized change ideas and lessons learned to inform future ABC program design and scale. Focus groups were conducted in Kinyarwanda and French using a standardized guide by a focus group facilitator and two note-takers. Follow-up structured debriefs of the facilitator and note takers were conducted by a qualitative expert to extract key themes of the discussions.

### Analysis

#### Collaborative implementation

We assessed the fidelity and completeness of the ABC initiative implementation comparing key activities including mentor visit frequency, site participation and QI activities with the program design. Qualitative data on facilitators and challenges to ABC success were also collected through the focus groups described above.

#### HCW attitudes and practice of quality improvement

Learning session surveys were entered into a database using EpiInfo version 7.1.5. Individual surveys with < 20% of questions completed were classified as incomplete and excluded. Likert scale questions responses were converted into dichotomous variables (4 or 5 versus < 3). Descriptive statistics were used with significance testing using chi-squared or two-tailed t-tests to measure difference between the baseline (pre-learning session) and endpoint (Harvest Session) for dichotomous and continuous variables respectively. Missing responses to individual questions were excluded and results with a *p* <  0.05 were considered significant. Quantitative analyses were conducted in Stata v14 (College Station, TX: StataCorp LP).

#### Change package

Quantitative success of QI projects was defined as significant change (p <  0.05) from the mean facility baseline versus QI project endpoint for the targeted core indicator using two-tailed t-tests.

Qualitative success for QI projects was based on QI team priority matrix rankings of change ideas as noted above and on the thematic coding of the focus group discussions. Both deductive and inductive approaches were used to determine underlying themes. To reduce reporting bias, the codes extracted from the interviews were validated by an expert in ABC implementation (ABC mentor) and by an expert in qualitative analysis.

Qualitative and quantitative data were subsequently integrated to determine which QI projects and change ideas warranted inclusion in the change package using rules summarized in Table 2.

**Table 2:** Rules for Determining Change Package Inclusion

Rule 1: All QI projects (which may contain multiple change ideas) demonstrating significant, positive pre/post mean change are included in the change package.	
RULE 2: All QI projects that do not have data available for pre/post mean analysis (small ‘n’ or rare occurrence) but were identified through the priority matrix and subsequently verified by focus groups as important change ideas are included in the change package.	
RULE 3: QI projects which were identified by the priority matrix and focus group as high impact, but quantitative data is available and does not demonstrate impact are excluded from the change package.	
RULE 4: If there are QI projects (ANC, postnatal, delivery) that contain multiple change ideas, implemented simultaneously and leading to improvement in a core indicator, then qualitative data was used to determine the relative merits of the different change ideas introduced. If a given change idea was identified by focus group participants as low impact then this idea is excluded from the change package.	

## Results

During the collaboratives, learning sessions were conducted every 3–5 months with an average of 40 participants per session and 98% of all health facilities represented at each session. Each health facility received, on average, 0.76 mentorship visits per month (range 0.6–0.9), slightly less than the planned 1 visit per month. Clinical trainings were implemented on average every 12.5 months with > 92% of facilities achieving the goal of retaining ≥2 trained nurses at the end of ABC (Table 3).

**Table 3:** ABC Learning Collaborative vs Typical Learning Collaborative Implementation Comparison [7, 10, 17]

Characteristic	ABC Learning collaborative	Common Learning Collaborative Design Components
Focus	Neonatal Mortality	Single clinical subject
# and type of Indicators addressed	8 indicators focused on drivers of neonatal mortality	1–9 indicators focused clinical subject
Duration of intervention	18 months	12–24 months
# people on QI team	3–4 people	3–7 people
Composition of QI team	Community health worker, nurse, data manager +/−leadership	Multi-professional team
Learning Session Characteristics	2 days	1–3 days
	4 learnings sessions + Harvest Session	> 2 sessions
	Every 3–5 months	every 4–6 months
Average % health facilities represented at all LS	98%	N/A
Average total # LS attendees	40.7	30-40people
Inclusion of Clinical Training or Skills Building	every 12.5 months	not standard
% HC with at least 2 nurses trained in newborn training package	> 92% throughout collaborative	not standard
Frequency of assessment for essential equipment	every 3 months	not standard
Mentor Characteristics	nurses with QI training	QI experts
Average mentor visits per facility per month	0.76 visits/month	Mentoring should occur between Learning Sessions
Monthly mentor visit content	QI project and QI skills mentorship	QI project and QI skills mentorship.
	Clinical skills mentorship and observation checklists for [1] Labor and delivery [2] neonatal care and/or [3] postpartum care	not standard
	routine equipment/commodities assessment and support	not standard

### HCW attitudes and practice of quality improvement

Self-reported QI capacity increased significantly by the end of the collaborative. This included increase in self-rated QI knowledge (37% vs 89%, *p* <  0.001), confidence (47% vs 89%, p <  0.001) and leadership (59% vs 91%, p <  0.001) (Table 4). QI team members also reported an increase in being asked for advice to improve neonatal care at the health facility (64 to 93%, p <  0.001).

**Table 4:** Results from Participant Survey Pre and Post Learning Collaborative^a, b^

	Pre-ABC LC	Post-ABC LC	*P*-value
Number participants	71	67	
Percent rating QI Knowledge and Confidence 4 or 5 *scale 1 (little/none) to 5 (very/extremely)*			
QI Knowledge	24/65 (37%)	58/65 (89%)	< 0.001
Confidence in helping to improve quality at your clinic or department/district	30/64 (47%)	58/65 (89%)	< 0.001
Confidence in leading QI at your clinic	34/58 (59%)	61/67 (91%)	< 0.001
Percent rating Leadership as very Interested in QI (scale *1 (not at all) to 3 (very interested)).*			
Facility management interest in measuring and improving quality for neonatal patients	43/68 (63%)	63/66 (95%)	< 0.001
Facility management interest in hearing ideas from you and other staff for QI?	38/67 (57%)	43/64 (67%)	0.2
Participant motivation at work (percent always or often) *(scale never, sometimes, often always)*			
My work is rewarding	17/63 (29%)	18/63 (27%)	0.84
My work is stressful	4/65 (6%)	3/63 (5%)	1.0
I feel emotionally drained by my work	53/63 (84%)	43/60 (72%)	0.09
I feel isolated in my work	4/66 (6%)	0/64 (0%)	1.0
I can help my clients	62/65 (97%)	65/67 (98%)	0.62
I have confidence in my skills	63/64 (98%)	61/63 (97%)	0.62
I am motivated to perform well in my job	42/67 (63%)	35/63 (55%)	0.35
Working Environment (percent very good or Excellent) *(scale poor, fair, good, very good, excellent)*			
Maternity/neonatal services at my clinic/hospital are excellent	31/59 (53%)	23/64 (36%)	0.06
I have enough basic clinical equipment and supplies to provide good care and services to patients	41/62 (66%)	51/61 (84%)	0.03
I have adequate support and information to help make clinical decisions	53/67 (79%)	41/57 (72%)	0.35
There is enough space for me to be able to provide care for the patients and ensure privacy	45/66 (68%)	44/61 (72%)	0.42
The clinic has a very good environment (friendliness, teamwork, respect, lack of chaos)	44/63 (70%)	47/63 (75%)	0.79
Peer to Peer learning	N (% yes)	N (% yes)	
Since the last LS, have you heard any ideas or asked for help from the other participating HC/hospitals?	17/47 (36%)	40/60 (66%)	0.024
Team work			
Have you been involved in activities that look at the quality of neonatal care and work to improve problems at your site?	36/64 (56%)	55/63 (87%)	< 0.001
Do staff members work together to improve quality of care for neonatal patients?	59/63 (93%)	61/64 (95%)	0.72
Have you been asked for your input how to improve neonatal care?	43/67 (64%)	59/63 (93%)	< 0.001
Data Utilization			
Have you seen or used any routine reports for measuring the quality of care?	32/63 (50%)	37/62 (59%)	0.32
Identified barriers to delivery of quality care at their site (Yes/No)	*n* = 71	*N* = 67	
High number of patients	29 (40%)	34 (50%)	0.24
Number of staff	30 (42%)	27 (40%)	0.32
Social or economic problems in patients’ lives (getting to clinic, food, etc)	30 (42%)	21 (31%)	0.18
Training of staff	28 (39%)	14 (20%)	0.018
Communication between providers and patients	15 (21%)	9 (13%)	0.23
The staff does not work together as a team	14 (19%)	5 (7%)	0.03
Complexity of care (severity of illness, complicated pregnancy, etc)	8 (11%)	5 (7%)	0.56
Clinic flow	7 (9%)	6 (8%)	0.86

^a^ “Pre” data obtained at the start of the first learning session. “Post” data obtained at the start of the “Harvest Session”

^b^ Missing data for a specific question were excluded

Other significant improvements included QI team member engagement in activities to measure the quality of neonatal care (56 to 87%, p <  0.001) and peer-to-peer learning (36 to 66%, *p* = 0.024). Surveys showed no significant change in motivation at work or use of routine reports (50 to 59%, *p* = 0.32).

Reported leadership interest in measuring and improving quality of care increased during ABC (63 to 95%, p <  0.001), although leadership interest in hearing health care worker input on QI remained unchanged (57 to 67% *p* = 0.2).

In addition to self-reported increases in peer collaboration, 61% of change concepts from the change package were implemented successfully by more than one facility. Focus group discussions revealed that the project also fostered intra-facility collaborations: “*through [QI teams] implementing their QI projects, it made more services within the health facilities develop their own projects.”* This led to the spread of QI beyond ABC core indicators to lab services, pharmacy, vaccination, family planning and vitamin K administration.

Perception of availability of adequate equipment to provide care and services increased (66 to 84%, *p* = 0.03). However, although perception of training as a barrier improved (39 to 20% *p* = 0.018) the top four reported barriers to quality care delivery remained unchanged, including: high patient volume, inadequate staffing, socioeconomic challenges of patients, and staff training.

### ABC change package

Facilities initiated a total of 52 QI projects spanning all 3 care domains, testing 118 change ideas. Of the ideas tested, 63% were in ANC, 27% were in delivery management, and 10% were in PNC (Table 5). Six of the eight core indicators were addressed. No projects explicitly address asphyxia (which was covered during clinical trainings) and due to changes in documentation, QI projects promoting placing newborns skin-to-skin with mothers immediately after delivery which were implemented early in ABC were not captured by mentors in the QI tracking templates. Forty-six (38.9%) change ideas were determined to be high impact through quantitative or qualitative criteria and were summarized into 17 change concepts for the change package (Table 6).

**Table 5:** Total QI Projects, Change Ideas and Care Domain Associated High Impact Themes

	Antenatal Care^a^	Delivery Care	Postnatal Care
N (%) QI Projects^b^	23 (44%)	17 (33%)	12 (23%)
N (%) Change Ideas^c^	74 (63%)	32 (27%)	12 (10%)
Change package associate themes			
Integration of services	✔	✔	✔
Teamwork & communication	✔	✔	✔
Mentorship & Clinical Teaching	✔	✔	✔
Data utilization	✔	✔	✔
Importance of leadership	✔		✔
Essential equipment	✔	✔	

^**a**^ Check mark indicates theme coded focus group debrief

^b^ QI Project: Goal set for improvement in targeted care area

^c^Change Idea: Specific intervention planned to achieve QI Project goal

**Table 6:** All Babies Count Change Package

Quality Indicator Targeted	Care Gap	Change Concept	Related Qualitative theme from focus groups	Facilities successfully implementing
QI PROJECT: IMPROVING ANTENATAL CARE SERVICES (# Facilities attempting n = 23)				
4 Antenatal care visits	Low 4 ANC visit completion because women miss 1st ANC appointment	Test women for pregnancy in all departments and transfer for ANC enrollment or same day ANC care if pregnant.	Integration of services Teamwork & Communic-ation	7
	Low community awareness of importance of ANC visits	Increase community awareness of ANC importance by educating women in waiting rooms of the health facility	Teamwork & Communic-ation	2
		Engage health center leadership to provide ANC care and demonstrate importance of ANC to the community.	Importance of Leadership	1
		Engage community leadership to help CHWs emphasize the importance of antenatal care in monthly community meetings	Importance of Leadership	1
	Health Centers too far for women to reach	Decentralize ANC services to Health Posts on a regular basis		4
	No mechanism to follow up women who miss appointments	Make (a) a filing system of medical records or (b) a register modification to facilitate identification of women who miss appointments for outreach by CHWs	Teamwork & Communica-tion	3
	No mechanism to remind women of up-coming appointments	Have CHWs remind women who have upcoming appointments.	Teamwork & Communica-tion	1
	Women cannot attend ANC clinic on the day offered	Offer ANC care at the health center more frequently (ranges from 2 times per week to daily) and at times that coordinate with community activities (such as market day)		4
	Women don’t come for ANC because partner is not available.	See women for 1st ANC and send her with invitation for her partner to attend following visit	Mentorship &Training	2
QI PROJECT: IMPROVING DELIVERY CARE SERVICES (# Facilities attempting n = 17)				
Time to C-section (District Hospitals only)	Poor communication and coordination between maternity and neonatal services	Set aside a regularly scheduled time for collaboration between neonatal and maternity services	Teamwork & Communication	2
Appropriate administration of steroids & antibiotics for preterm labor management	Low case identification of women in active preterm labor	Define scope of problem at health center by comparing preterm infants recorded versus women in preterm labor identified	Data utilization	5
		Refresher trainings for staff on calculation of gestational age and management of PPROM in order to improve recognition of labor complications & management	Mentorship & Training	4
	Medical Staff forget that steroids and/or antibiotics may be indicated in preterm birth	Modify existing maternity registers to prompt appropriate management	Integration of services Teamwork & Communication	2
	Antibiotics not given because not available	Have nurses proactively checking on supply of steroids available.	Essential equipment	1
Facility-based delivery	Women not deliver at the facility because inadequate anticipatory planning	Assist mothers with anticipatory planning of items to have prepared to bring for delivery at their 3rd ANC appointment	Teamwork & Communication	1
QI PROJECT: IMPROVING POSTNATAL CARE SERVICES (# Facilities attempting n = 12)				
Checking infants for Danger Signs within 24 hours	Low maternal knowledge & nurse vigilance in checking for neonatal danger signs	Make checking for danger signs part of the maternity register and assign the filling of the register as a daily nursing responsibly to prompt staff to educate the mother and check the newborn for Danger Signs	Integration of services	5
	Short hospital stays (often same day discharge) preclude staff from checking danger signs	When women come to get child’s BCG vaccination makes sure they are also screened for neonatal danger signs	Integration of services	1

Successful change concepts included improving access to and convenience of ANC services, improving community engagement and awareness of ANC service importance, and leadership engagement. Interventions targeting delivery included improving coordination of care to reduce time to caesarian section, preterm labor management included refresher trainings, collaborations with pharmacy to ensure stock of necessary medications and gestational age calculation training and verification. QI projects focused on PNC included danger sign recognition within 24 h of delivery and return for postnatal check-up after discharge. Successful PNC change concepts included patient register modification to act as a clinical reminder for HCW to check mother-baby dyad and integration of postnatal check-ups into routine vaccination services. Participants identified areas which could not be addressed by the ABC scope such as “*retaining patients for 72 hours* [for PNC care] *was difficult and unsuccessful because of high volume of patients. They don’t have enough rooms, staff, or beds.”*

Table 5 describes six general themes of high impact projects identified by focus group participants. One theme was the importance of the integration of services. For example, an intervention to improve ANC visit attendance was reported as working well because it “*integrate[d] the tracking of women who tested positive for pregnancy when they came in to other services.”* Similarly, PNC service attendance was improved by taking a “*strategy to attract more mothers* [to post-natal services by getting] *them when they came for vaccination clinics.”* Projects were noted to be more sustainable when there was integration, “*... between health system, local leaders and community health workers.”*

Teamwork and communication were also identified as important themes for success across all care domains. “[*Teamwork*] *helps make a project be successful. Working together and learning from each other are what make projects successful.”* Leadership support emerged as another key element to high impact project implementation with debriefs mentioning that “*Local leadership collaboration allowed for QI projects to be successful.”* Integrated clinical and QI mentorship were also identified as important facilitators of high impact QI projects. “*The mentors would come to health facilities while they worked and correct them on the job. They would see where they needed more tools or supplies…this would keep the participants active and working on their QI projects.”*

Clinical training where the “*staff gained skills through (ABC) to implement QI projects”* on key practices between or during learning sessions was also reported as valuable. Availability of essential equipment was also noted to be key to the success, as “*equipment and materials were a problem at in the beginning of the QI projects but then they received the materials from ABC”.* Finally, participants reported the importance of data utilization to QI efforts “*[the ability to do] data monitoring in order to track their progress in implementation”* was key to the execution of high impact projects.

## Discussion

The ABC initiative successfully integrated clinical and QI mentorship with a learning collaborative and targeted training and equipment support designed to improve newborn care in district health care facilities in rural Rwanda. ABC was implemented with strong fidelity and acceptability to participants, resulting in wide-spread QI activities at the health center and district hospital levels. The initiative also increased district-wide QI capacity and peer-to-peer learning. The program success was accomplished by engaging individuals and teams through mentorship and learning sessions to improve individuals’ and system’s ability to create, implement and spread QI projects across the district.

Our finding that a multi-faceted intervention centered around a learning collaborative was an effective model in building QI capacity and practice improvement was similar to Franco’s findings from a review of learning collaboratives in resource limited settings [5]. Quality delivery of evidence-based interventions is dependent on healthcare worker skills and availability of supplies, especially in resource limited settings [19–21]. However, studies have found that addressing facility readiness in isolation does not lead to improved delivery of high quality care. ABC’s explicit attention to essential equipment at the start of the project and baseline training in essential care practices to address potential barriers of knowledge and skills was identified as contributing to the successful implementation and acceptability of ABC in addition to the ongoing mentoring and QI. These findings emphasize the importance of comprehensive interventions, such as ABC, which have the ability to address facility readiness as well as continuous learning through on-going training and effective supportive supervision or mentoring [22–26].

The success of ABC in building QI capacity was evident in the reported increase in QI confidence and the range and scope of change ideas implemented. Additionally, work to increase data use for performance measurement, a key step of PDSA cycles, was emphasized in some of the change ideas. For example, maternity and neonatal registers were compared to identify cases of inadequate preterm labor screening, and stock checks for essential medications were integrated into routine activities.

Change packages generated through collaboratives are an important product for spreading change [27]. However, there is little consistency or description of how change packages are created [6] with previous collaboratives in LMIC settings describing a range of methods. *Project Fives Alive!* in Ghana took a strictly quantitative approach using run chart rules [28]. Others, such as projects supported by the USAID ASSIST [12, 29, 30] describe a more mixed-methods approach to change package creation. We chose a mixed-methods approach to incorporate front line HCWs and identify projects which may not be seen through quantitative analysis, but which were feasible, high impact and applicable to the setting. This addition of qualitative data was particularly relevant when events were rare so statistical significance was unlikely to be reached, yet had potentially critical impact on patient outcomes (i.e. antibiotics for prolonged rupture of membranes), or the number of data points too few to use run charts or statistical process control charts, other methods commonly used in QI [31].

Our study had a number of limitations. Changes in capacity and peer-to-peer learning were solely based on HCW self-report. We also could not analyze change at the individual level since some providers’ attending of the learning sessions changes over time due to staff turnover. We also did not have any counterfactual evidence, so could not prove that changes in QI capacity and activities were due to the ABC intervention nor which components of ABC were most important in driving those changes. However, we do know that no other independent neonatal-focused or other QI initiatives were newly active in the districts during ABC.

The quality of the data and documentation likely improved over the course of ABC implementation, potentially contributing to some of the measured improvement associated with change ideas. We also did not have sufficient data subgroups at the change idea level for the application of run chart rules. Therefore, we used pre-post means of project level measures and included qualitative assessment of change ideas. Impact on quality and cost-effectiveness are not included and will be published separately.

## Conclusion

Incorporating clinical mentorship and facility readiness support in the targeted areas into district-wide learning collaboratives was a feasible and effective strategy to support the development of a culture and capacity for QI in rural districts in Rwanda. Including evaluation of implementation contributed to the ability to proceed with scale-up of the intervention including timely application of the change package in Rwanda. Final analysis of ABC impact on care outcomes and sustainability one year post-ABC is underway.

## Abbreviations

ABC: All Babies Count
ANC: Antenatal Care
HCW: Healthcare Worker
HMIS: Health Management Information System
MOH: Ministry of Health
PNC: Postnatal Care
QI: Quality Improvement
